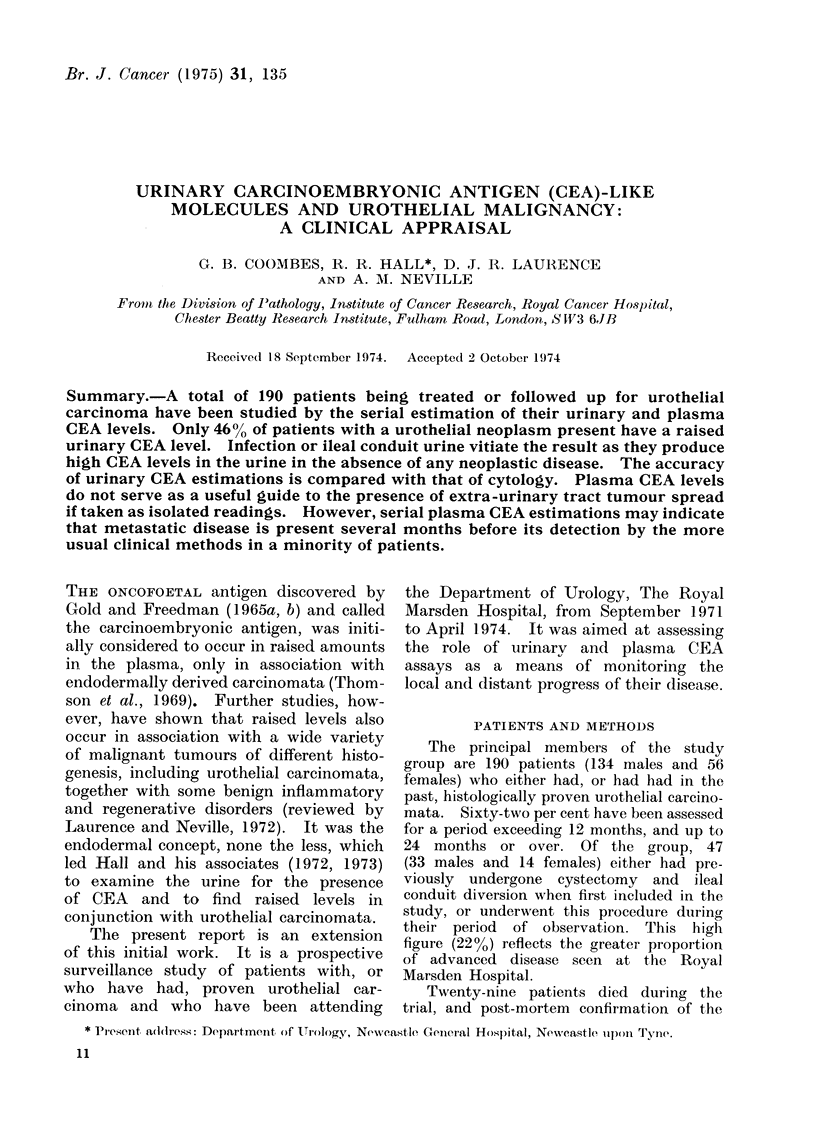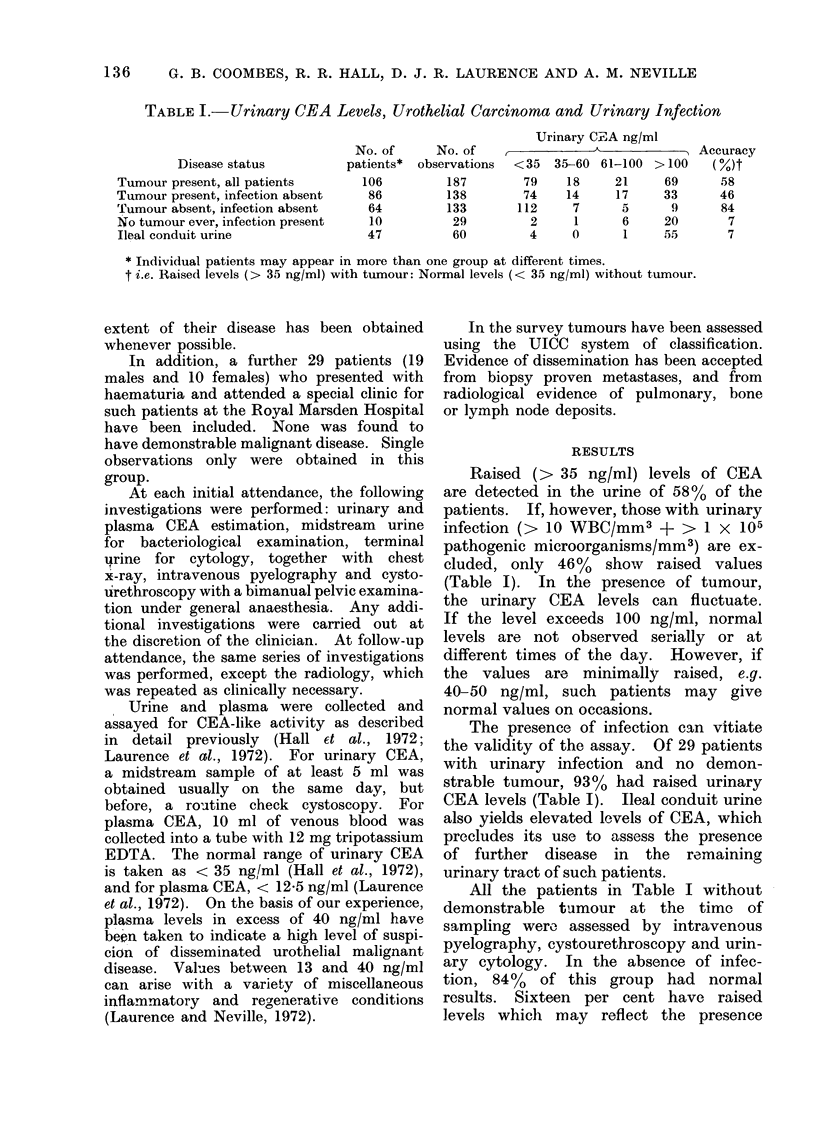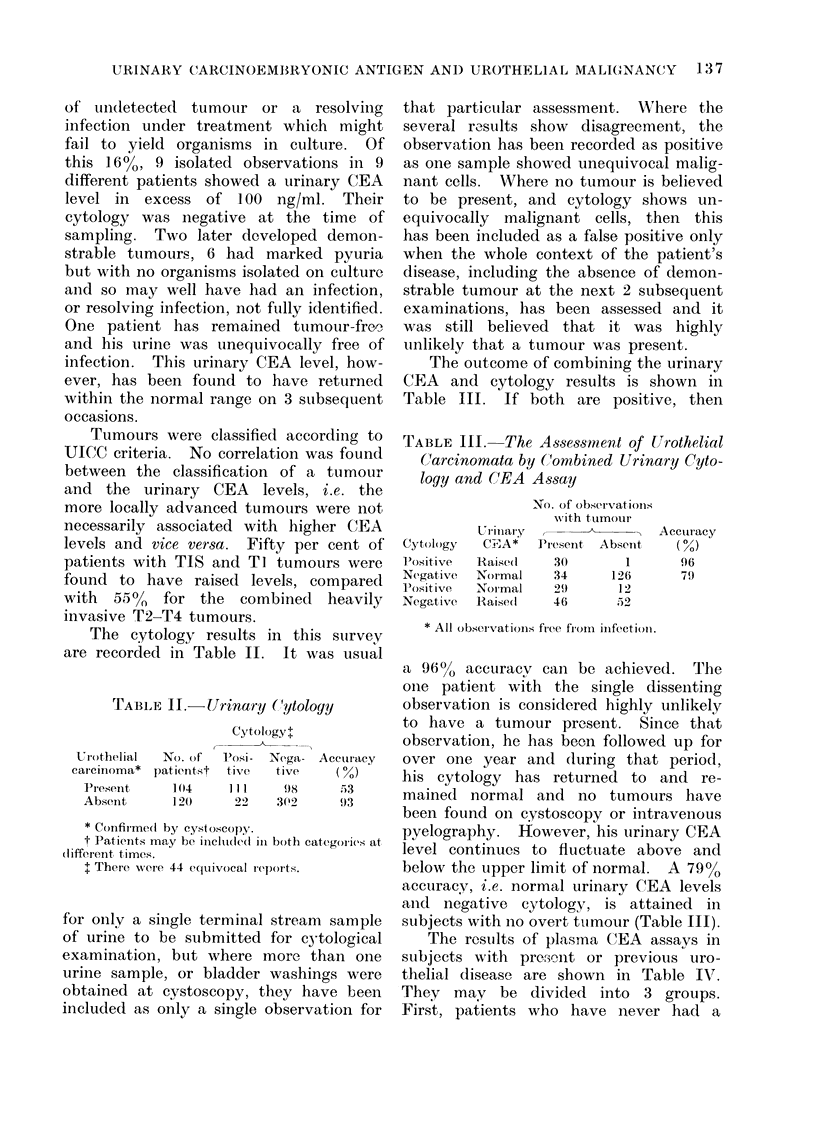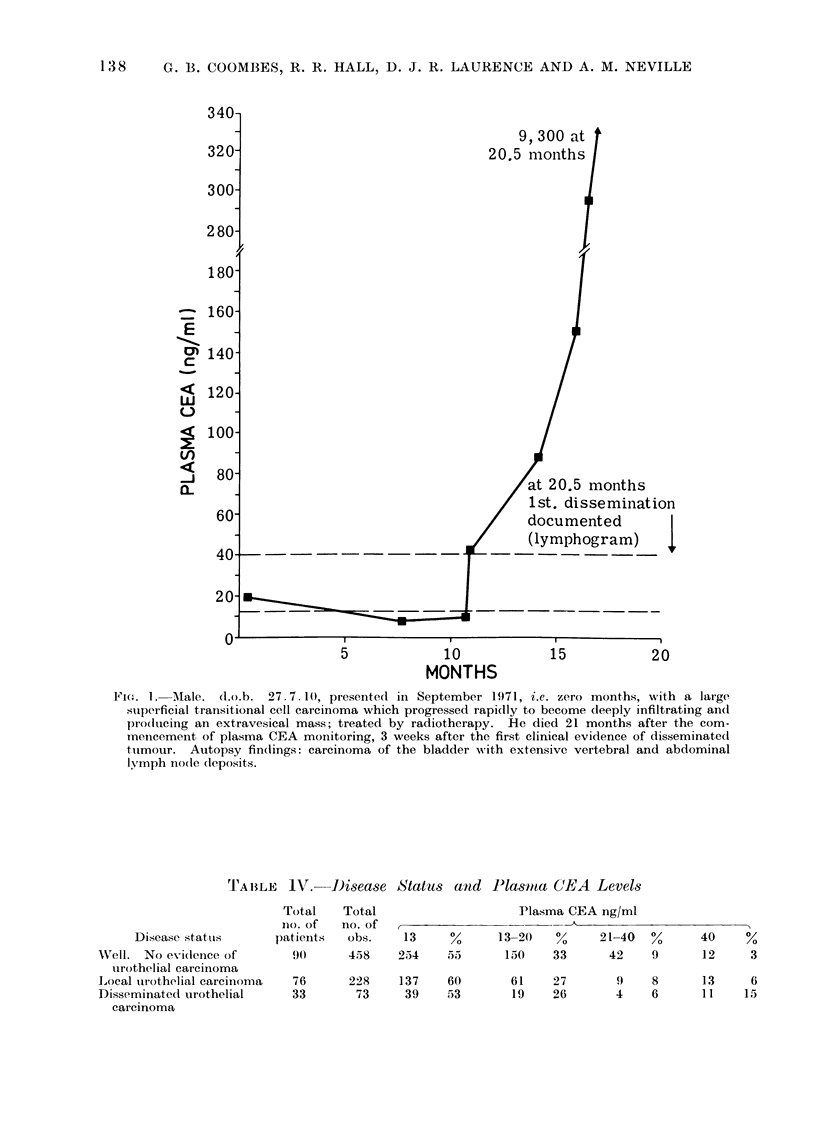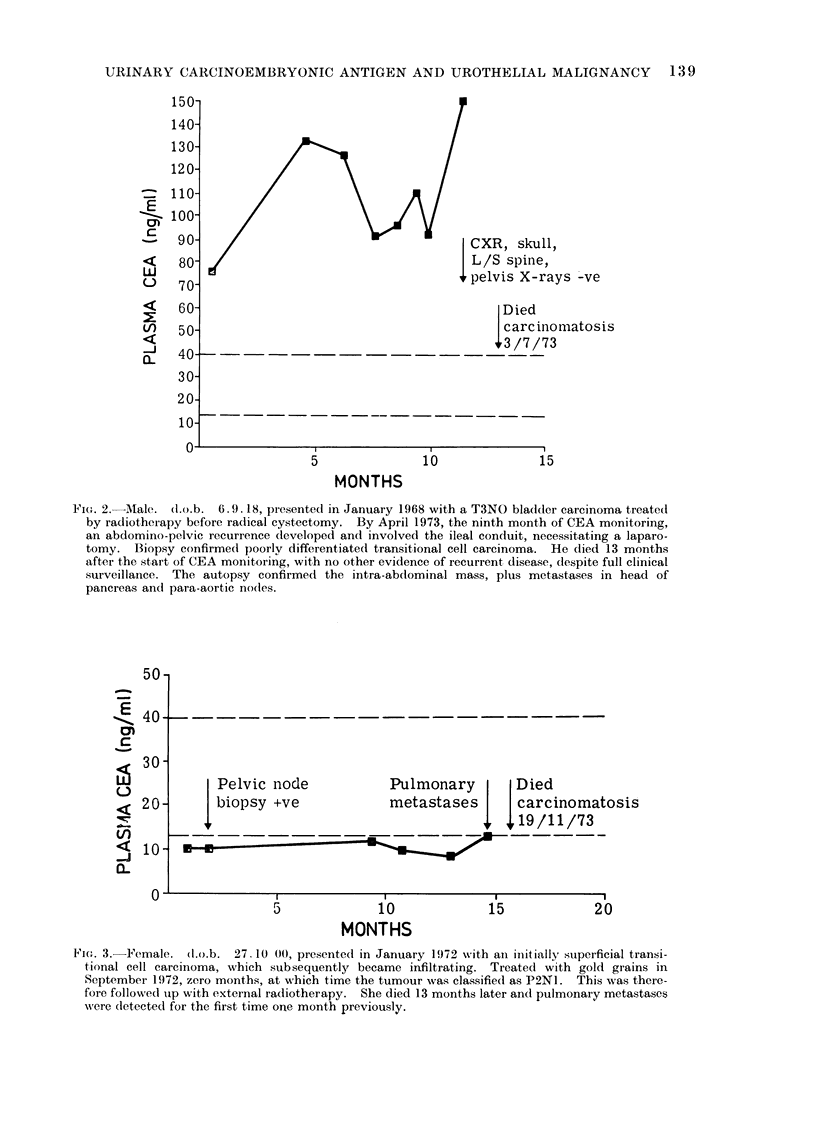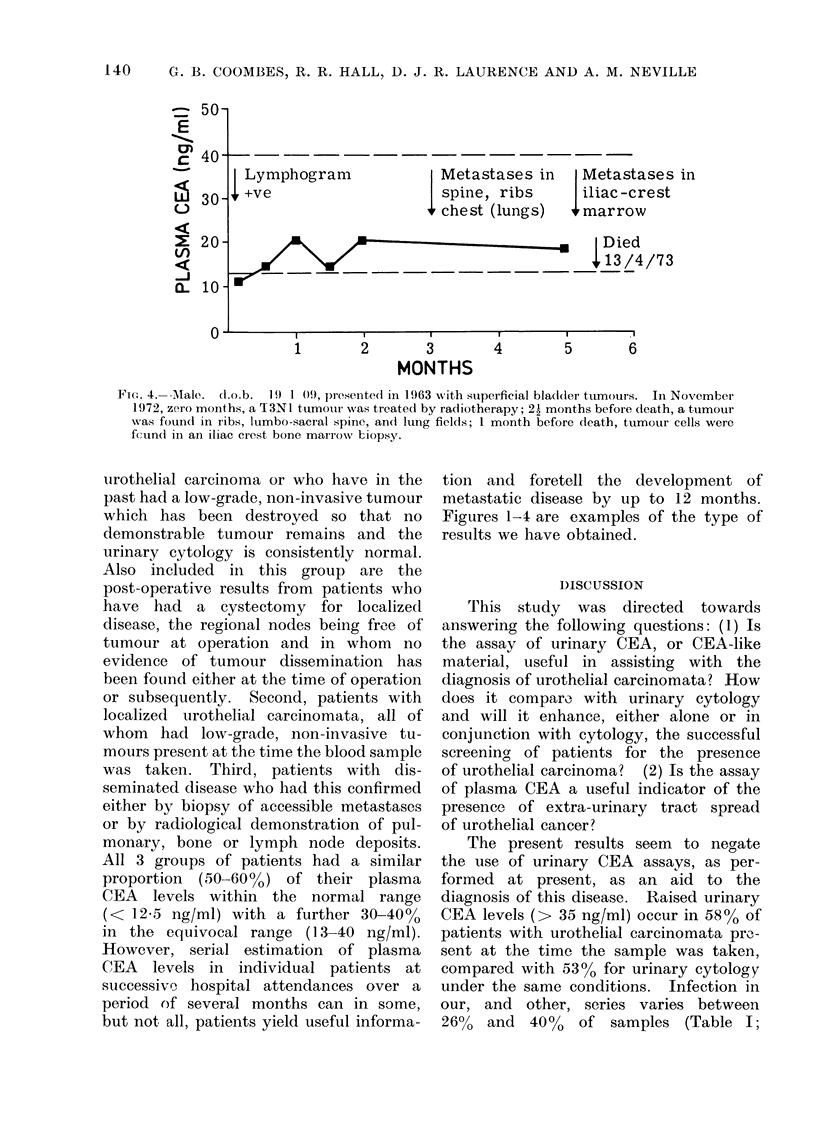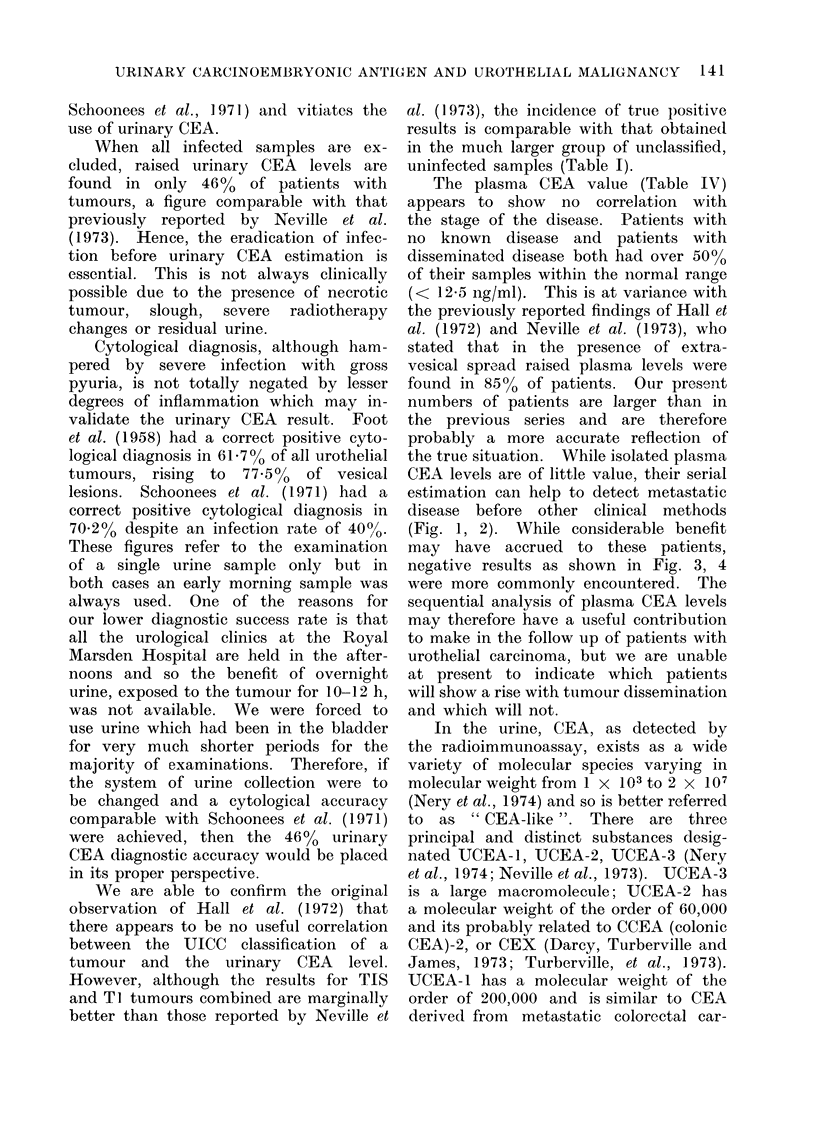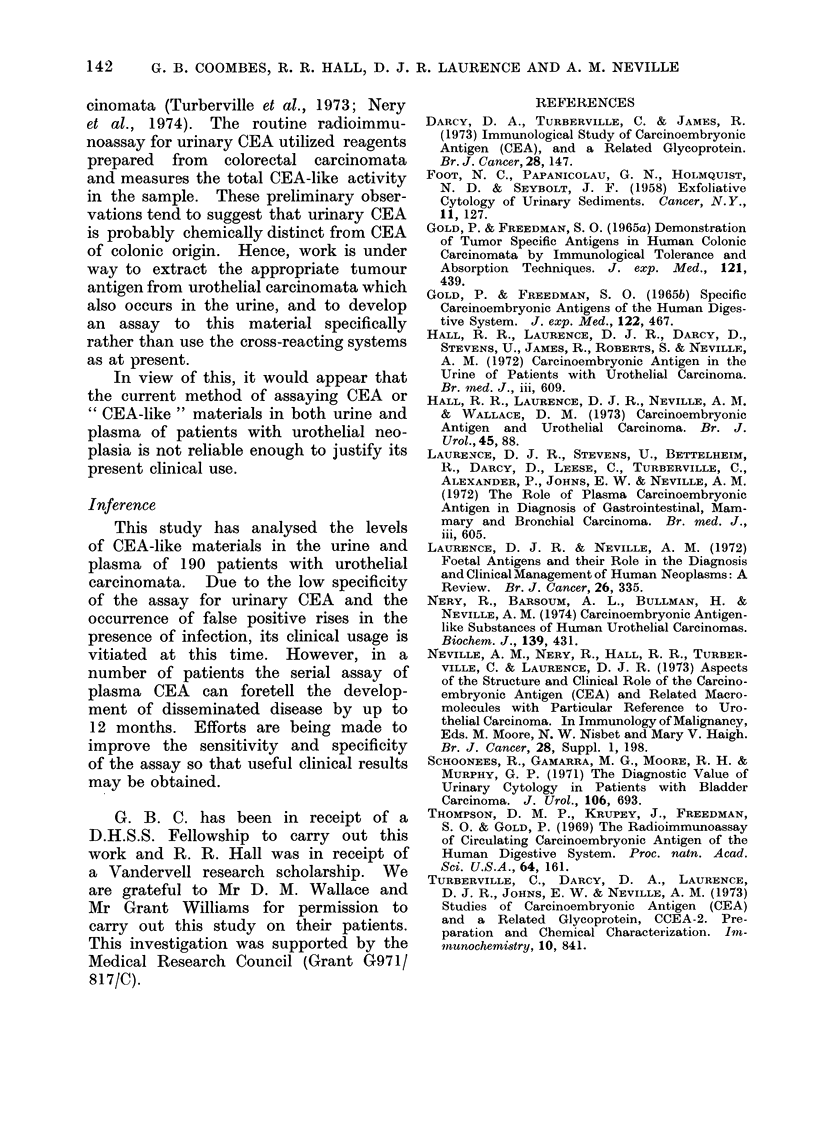# Urinary carcinoembryonic antigen (CEA)-like molecules and urothelial malignancy: a clinical appraisal.

**DOI:** 10.1038/bjc.1975.18

**Published:** 1975-02

**Authors:** G. B. Coombers, R. R. Hall, J. R. Laurence, A. M. Neville

## Abstract

A total of 190 patients being treated or followed up for urothelial carcinoma have been studied by the serial estimation of their urinary and plasma CEA levels. Only 46% of patients with a urothelial neoplasm present have a raised urinary CEA level. Infection or ileal conduit urine vitiate the result as they produce high CEA levels in the urine in the absence of any neoplastic disease. The accuracy of urinary CEA estimations is compared with that of cytology. Plasma CEA levels do not serve as a useful guide to the presence of extra-urinary tract tumour spread if taken as isolated readings. However, serial plasma CEA estimations may indicate that metastatic disease is present several months before its detection by the more usual clinical methods in a minority of patients.


Br. J. Cancer (1975) 31, 135

### URINARY CARCINOEMBRYONIC ANTIGEN (CEA)-LIKE MOLECULES AND UROTHELIAL MALIGNANCY: A CLINICAL APPRAISAL

G. B. COOMBES, R. R. HALL*, D. J. R. LAURENCE AND A. M. NEVILLE

*From the Division of Pathology, Institute of Cancer Research, Royal Cancer Hospital, Chester Beatty Research Institute, Fulham Road, London, SW3 6JB*

Received 18 September 1974. Accepted 2 October 1974

**Summary.—A total of 190 patients being treated or followed up for urothelial carcinoma have been studied by the serial estimation of their urinary and plasma CEA levels. Only 46% of patients with a urothelial neoplasm present have a raised urinary CEA level. Infection or ileal conduit urine vitiate the result as they produce high CEA levels in the urine in the absence of any neoplastic disease. The accuracy of urinary CEA estimations is compared with that of cytology. Plasma CEA levels do not serve as a useful guide to the presence of extra-urinary tract tumour spread if taken as isolated readings. However, serial plasma CEA estimations may indicate that metastatic disease is present several months before its detection by the more usual clinical methods in a minority of patients.**

THE ONCOFOETAL antigen discovered by Gold and Freedman (1965*a*, *b*) and called the carcinoembryonic antigen, was initially considered to occur in raised amounts in the plasma, only in association with endodermally derived carcinomata (Thomson *et al.*, 1969). Further studies, however, have shown that raised levels also occur in association with a wide variety of malignant tumours of different histogenesis, including urothelial carcinomata, together with some benign inflammatory and regenerative disorders (reviewed by Laurence and Neville, 1972). It was the endodermal concept, none the less, which led Hall and his associates (1972, 1973) to examine the urine for the presence of CEA and to find raised levels in conjunction with urothelial carcinomata.

The present report is an extension of this initial work. It is a prospective surveillance study of patients with, or who have had, proven urothelial carcinoma and who have been attending the Department of Urology, The Royal Marsden Hospital, from September 1971 to April 1974. It was aimed at assessing the role of urinary and plasma CEA assays as a means of monitoring the local and distant progress of their disease.

### PATIENTS AND METHODS

The principal members of the study group are 190 patients (134 males and 56 females) who either had, or had had in the past, histologically proven urothelial carcinomata. Sixty-two per cent have been assessed for a period exceeding 12 months, and up to 24 months or over. Of the group, 47 (33 males and 14 females) either had previously undergone cystectomy and ileal conduit diversion when first included in the study, or underwent this procedure during their period of observation. This high figure (22%) reflects the greater proportion of advanced disease seen at the Royal Marsden Hospital.

Twenty-nine patients died during the trial, and post-mortem confirmation of the

* Present address: Department of Urology, Newcastle General Hospital, Newcastle upon Tyne.

TABLE I.—*Urinary CEA Levels, Urothelial Carcinoma and Urinary Infection*

| Disease status | No. of patients* | No. of observations | Urinary CEA ng/ml <35 | 35–60 | 61–100 | >100 | Accuracy (%)† |
|---|---|---|---|---|---|---|---|
| Tumour present, all patients | 106 | 187 | 79 | 18 | 21 | 69 | 58 |
| Tumour present, infection absent | 86 | 138 | 74 | 14 | 17 | 33 | 46 |
| Tumour absent, infection absent | 64 | 133 | 112 | 7 | 5 | 9 | 84 |
| No tumour ever, infection present | 10 | 29 | 2 | 1 | 6 | 20 | 7 |
| Ileal conduit urine | 47 | 60 | 4 | 0 | 1 | 55 | 7 |

* Individual patients may appear in more than one group at different times.
† *i.e.* Raised levels (> 35 ng/ml) with tumour: Normal levels (< 35 ng/ml) without tumour.

extent of their disease has been obtained whenever possible.

In addition, a further 29 patients (19 males and 10 females) who presented with haematuria and attended a special clinic for such patients at the Royal Marsden Hospital have been included. None was found to have demonstrable malignant disease. Single observations only were obtained in this group.

At each initial attendance, the following investigations were performed: urinary and plasma CEA estimation, midstream urine for bacteriological examination, terminal urine for cytology, together with chest x-ray, intravenous pyelography and cysto-urethroscopy with a bimanual pelvic examination under general anaesthesia. Any additional investigations were carried out at the discretion of the clinician. At follow-up attendance, the same series of investigations was performed, except the radiology, which was repeated as clinically necessary.

Urine and plasma were collected and assayed for CEA-like activity as described in detail previously (Hall *et al.*, 1972; Laurence *et al.*, 1972). For urinary CEA, a midstream sample of at least 5 ml was obtained usually on the same day, but before, a routine check cystoscopy. For plasma CEA, 10 ml of venous blood was collected into a tube with 12 mg tripotassium EDTA. The normal range of urinary CEA is taken as < 35 ng/ml (Hall *et al.*, 1972), and for plasma CEA, < 12·5 ng/ml (Laurence *et al.*, 1972). On the basis of our experience, plasma levels in excess of 40 ng/ml have been taken to indicate a high level of suspicion of disseminated urothelial malignant disease. Values between 13 and 40 ng/ml can arise with a variety of miscellaneous inflammatory and regenerative conditions (Laurence and Neville, 1972).

In the survey tumours have been assessed using the UICC system of classification. Evidence of dissemination has been accepted from biopsy proven metastases, and from radiological evidence of pulmonary, bone or lymph node deposits.

### RESULTS

Raised (> 35 ng/ml) levels of CEA are detected in the urine of 58% of the patients. If, however, those with urinary infection (> 10 WBC/mm$^3$ + > 1 × 10$^5$ pathogenic microorganisms/mm$^3$) are excluded, only 46% show raised values (Table I). In the presence of tumour, the urinary CEA levels can fluctuate. If the level exceeds 100 ng/ml, normal levels are not observed serially or at different times of the day. However, if the values are minimally raised, *e.g.* 40–50 ng/ml, such patients may give normal values on occasions.

The presence of infection can vitiate the validity of the assay. Of 29 patients with urinary infection and no demonstrable tumour, 93% had raised urinary CEA levels (Table I). Ileal conduit urine also yields elevated levels of CEA, which precludes its use to assess the presence of further disease in the remaining urinary tract of such patients.

All the patients in Table I without demonstrable tumour at the time of sampling were assessed by intravenous pyelography, cystourethroscopy and urinary cytology. In the absence of infection, 84% of this group had normal results. Sixteen per cent have raised levels which may reflect the presence of undetected tumour or a resolving infection under treatment which might fail to yield organisms in culture. Of this 16%, 9 isolated observations in 9 different patients showed a urinary CEA level in excess of 100 ng/ml. Their cytology was negative at the time of sampling. Two later developed demonstrable tumours, 6 had marked pyuria but with no organisms isolated on culture and so may well have had an infection, or resolving infection, not fully identified. One patient has remained tumour-free and his urine was unequivocally free of infection. This urinary CEA level, however, has been found to have returned within the normal range on 3 subsequent occasions.

Tumours were classified according to UICC criteria. No correlation was found between the classification of a tumour and the urinary CEA levels, *i.e.* the more locally advanced tumours were not necessarily associated with higher CEA levels and *vice versa.* Fifty per cent of patients with TIS and T1 tumours were found to have raised levels, compared with 55% for the combined heavily invasive T2–T4 tumours.

The cytology results in this survey are recorded in Table II. It was usual

TABLE II.—*Urinary Cytology*

| Urothelial carcinoma* | No. of patients† | Cytology‡ Positive | Negative | Accuracy (%) |
|---|---|---|---|---|
| Present | 104 | 111 | 98 | 53 |
| Absent | 120 | 22 | 302 | 93 |

* Confirmed by cystoscopy.

† Patients may be included in both categories at different times.

‡ There were 44 equivocal reports.

for only a single terminal stream sample of urine to be submitted for cytological examination, but where more than one urine sample, or bladder washings were obtained at cystoscopy, they have been included as only a single observation for that particular assessment. Where the several results show disagreement, the observation has been recorded as positive as one sample showed unequivocal malignant cells. Where no tumour is believed to be present, and cytology shows unequivocally malignant cells, then this has been included as a false positive only when the whole context of the patient's disease, including the absence of demonstrable tumour at the next 2 subsequent examinations, has been assessed and it was still believed that it was highly unlikely that a tumour was present.

The outcome of combining the urinary CEA and cytology results is shown in Table III. If both are positive, then

TABLE III.—*The Assessment of Urothelial Carcinomata by Combined Urinary Cytology and CEA Assay*

| Cytology | Urinary CEA* | No. of observations with tumour Present | Absent | Accuracy (%) |
|---|---|---|---|---|
| Positive | Raised | 30 | 1 | 96 |
| Negative | Normal | 34 | 126 | 79 |
| Positive | Normal | 29 | 12 | |
| Negative | Raised | 46 | 52 | |

* All observations free from infection.

a 96% accuracy can be achieved. The one patient with the single dissenting observation is considered highly unlikely to have a tumour present. Since that observation, he has been followed up for over one year and during that period, his cytology has returned to and remained normal and no tumours have been found on cystoscopy or intravenous pyelography. However, his urinary CEA level continues to fluctuate above and below the upper limit of normal. A 79% accuracy, *i.e.* normal urinary CEA levels and negative cytology, is attained in subjects with no overt tumour (Table III).

The results of plasma CEA assays in subjects with present or previous urothelial disease are shown in Table IV. They may be divided into 3 groups. First, patients who have never had a

FIG. 1.—Male. d.o.b. 27.7.10, presented in September 1971, *i.e.* zero months, with a large superficial transitional cell carcinoma which progressed rapidly to become deeply infiltrating and producing an extravesical mass; treated by radiotherapy. He died 21 months after the commencement of plasma CEA monitoring, 3 weeks after the first clinical evidence of disseminated tumour. Autopsy findings: carcinoma of the bladder with extensive vertebral and abdominal lymph node deposits.

TABLE IV.—*Disease Status and Plasma CEA Levels*

| Disease status | Total no. of patients | Total no. of obs. | Plasma CEA ng/ml | | | | | | | |
|---|---|---|---|---|---|---|---|---|---|---|
| | | | 13 | % | 13–20 | % | 21–40 | % | 40 | % |
| Well. No evidence of urothelial carcinoma | 90 | 458 | 254 | 55 | 150 | 33 | 42 | 9 | 12 | 3 |
| Local urothelial carcinoma | 76 | 228 | 137 | 60 | 61 | 27 | 9 | 8 | 13 | 6 |
| Disseminated urothelial carcinoma | 33 | 73 | 39 | 53 | 19 | 26 | 4 | 6 | 11 | 15 |

FIG. 2.—Male. d.o.b. 6.9.18, presented in January 1968 with a T3NO bladder carcinoma treated by radiotherapy before radical cystectomy. By April 1973, the ninth month of CEA monitoring, an abdomino-pelvic recurrence developed and involved the ileal conduit, necessitating a laparotomy. Biopsy confirmed poorly differentiated transitional cell carcinoma. He died 13 months after the start of CEA monitoring, with no other evidence of recurrent disease, despite full clinical surveillance. The autopsy confirmed the intra-abdominal mass, plus metastases in head of pancreas and para-aortic nodes.

FIG. 3.—Female. d.o.b. 27.10 00, presented in January 1972 with an initially superficial transitional cell carcinoma, which subsequently became infiltrating. Treated with gold grains in September 1972, zero months, at which time the tumour was classified as P2N1. This was therefore followed up with external radiotherapy. She died 13 months later and pulmonary metastases were detected for the first time one month previously.

FIG. 4.—Male. d.o.b. 19 1 09, presented in 1963 with superficial bladder tumours. In November 1972, zero months, a T3N1 tumour was treated by radiotherapy; 2½ months before death, a tumour was found in ribs, lumbo-sacral spine, and lung fields; 1 month before death, tumour cells were found in an iliac crest bone marrow biopsy.

urothelial carcinoma or who have in the past had a low-grade, non-invasive tumour which has been destroyed so that no demonstrable tumour remains and the urinary cytology is consistently normal. Also included in this group are the post-operative results from patients who have had a cystectomy for localized disease, the regional nodes being free of tumour at operation and in whom no evidence of tumour dissemination has been found either at the time of operation or subsequently. Second, patients with localized urothelial carcinomata, all of whom had low-grade, non-invasive tumours present at the time the blood sample was taken. Third, patients with disseminated disease who had this confirmed either by biopsy of accessible metastases or by radiological demonstration of pulmonary, bone or lymph node deposits. All 3 groups of patients had a similar proportion (50–60%) of their plasma CEA levels within the normal range (< 12·5 ng/ml) with a further 30–40% in the equivocal range (13–40 ng/ml). However, serial estimation of plasma CEA levels in individual patients at successive hospital attendances over a period of several months can in some, but not all, patients yield useful information and foretell the development of metastatic disease by up to 12 months. Figures 1–4 are examples of the type of results we have obtained.

### DISCUSSION

This study was directed towards answering the following questions: (1) Is the assay of urinary CEA, or CEA-like material, useful in assisting with the diagnosis of urothelial carcinomata? How does it compare with urinary cytology and will it enhance, either alone or in conjunction with cytology, the successful screening of patients for the presence of urothelial carcinoma? (2) Is the assay of plasma CEA a useful indicator of the presence of extra-urinary tract spread of urothelial cancer?

The present results seem to negate the use of urinary CEA assays, as performed at present, as an aid to the diagnosis of this disease. Raised urinary CEA levels (> 35 ng/ml) occur in 58% of patients with urothelial carcinomata present at the time the sample was taken, compared with 53% for urinary cytology under the same conditions. Infection in our, and other, series varies between 26% and 40% of samples (Table I;

Schoonees *et al.*, 1971) and vitiates the use of urinary CEA.

When all infected samples are excluded, raised urinary CEA levels are found in only 46% of patients with tumours, a figure comparable with that previously reported by Neville *et al.* (1973). Hence, the eradication of infection before urinary CEA estimation is essential. This is not always clinically possible due to the presence of necrotic tumour, slough, severe radiotherapy changes or residual urine.

Cytological diagnosis, although hampered by severe infection with gross pyuria, is not totally negated by lesser degrees of inflammation which may invalidate the urinary CEA result. Foot *et al.* (1958) had a correct positive cytological diagnosis in 61·7% of all urothelial tumours, rising to 77·5% of vesical lesions. Schoonees *et al.* (1971) had a correct positive cytological diagnosis in 70·2% despite an infection rate of 40%. These figures refer to the examination of a single urine sample only but in both cases an early morning sample was always used. One of the reasons for our lower diagnostic success rate is that all the urological clinics at the Royal Marsden Hospital are held in the afternoons and so the benefit of overnight urine, exposed to the tumour for 10–12 h, was not available. We were forced to use urine which had been in the bladder for very much shorter periods for the majority of examinations. Therefore, if the system of urine collection were to be changed and a cytological accuracy comparable with Schoonees *et al.* (1971) were achieved, then the 46% urinary CEA diagnostic accuracy would be placed in its proper perspective.

We are able to confirm the original observation of Hall *et al.* (1972) that there appears to be no useful correlation between the UICC classification of a tumour and the urinary CEA level. However, although the results for TIS and T1 tumours combined are marginally better than those reported by Neville *et al.* (1973), the incidence of true positive results is comparable with that obtained in the much larger group of unclassified, uninfected samples (Table I).

The plasma CEA value (Table IV) appears to show no correlation with the stage of the disease. Patients with no known disease and patients with disseminated disease both had over 50% of their samples within the normal range (< 12·5 ng/ml). This is at variance with the previously reported findings of Hall *et al.* (1972) and Neville *et al.* (1973), who stated that in the presence of extravesical spread raised plasma levels were found in 85% of patients. Our present numbers of patients are larger than in the previous series and are therefore probably a more accurate reflection of the true situation. While isolated plasma CEA levels are of little value, their serial estimation can help to detect metastatic disease before other clinical methods (Fig. 1, 2). While considerable benefit may have accrued to these patients, negative results as shown in Fig. 3, 4 were more commonly encountered. The sequential analysis of plasma CEA levels may therefore have a useful contribution to make in the follow up of patients with urothelial carcinoma, but we are unable at present to indicate which patients will show a rise with tumour dissemination and which will not.

In the urine, CEA, as detected by the radioimmunoassay, exists as a wide variety of molecular species varying in molecular weight from $1 \times 10^3$ to $2 \times 10^7$ (Nery *et al.*, 1974) and so is better referred to as "CEA-like". There are three principal and distinct substances designated UCEA-1, UCEA-2, UCEA-3 (Nery *et al.*, 1974; Neville *et al.*, 1973). UCEA-3 is a large macromolecule; UCEA-2 has a molecular weight of the order of 60,000 and its probably related to CCEA (colonic CEA)-2, or CEX (Darcy, Turberville and James, 1973; Turberville, *et al.*, 1973). UCEA-1 has a molecular weight of the order of 200,000 and is similar to CEA derived from metastatic colorectal carcinomata (Turberville *et al.*, 1973; Nery *et al.*, 1974). The routine radioimmunoassay for urinary CEA utilized reagents prepared from colorectal carcinomata and measures the total CEA-like activity in the sample. These preliminary observations tend to suggest that urinary CEA is probably chemically distinct from CEA of colonic origin. Hence, work is under way to extract the appropriate tumour antigen from urothelial carcinomata which also occurs in the urine, and to develop an assay to this material specifically rather than use the cross-reacting systems as at present.

In view of this, it would appear that the current method of assaying CEA or " CEA-like " materials in both urine and plasma of patients with urothelial neoplasia is not reliable enough to justify its present clinical use.

### *Inference*

This study has analysed the levels of CEA-like materials in the urine and plasma of 190 patients with urothelial carcinomata. Due to the low specificity of the assay for urinary CEA and the occurrence of false positive rises in the presence of infection, its clinical usage is vitiated at this time. However, in a number of patients the serial assay of plasma CEA can foretell the development of disseminated disease by up to 12 months. Efforts are being made to improve the sensitivity and specificity of the assay so that useful clinical results may be obtained.

G. B. C. has been in receipt of a D.H.S.S. Fellowship to carry out this work and R. R. Hall was in receipt of a Vandervell research scholarship. We are grateful to Mr D. M. Wallace and Mr Grant Williams for permission to carry out this study on their patients. This investigation was supported by the Medical Research Council (Grant G971/817/C).

### REFERENCES

DARCY, D. A., TURBERVILLE, C. & JAMES, R. (1973) Immunological Study of Carcinoembryonic Antigen (CEA), and a Related Glycoprotein. *Br. J. Cancer*, **28**, 147.

FOOT, N. C., PAPANICOLAU, G. N., HOLMQUIST, N. D. & SEYBOLT, J. F. (1958) Exfoliative Cytology of Urinary Sediments. *Cancer, N.Y.*, **11**, 127.

GOLD, P. & FREEDMAN, S. O. (1965*a*) Demonstration of Tumor Specific Antigens in Human Colonic Carcinomata by Immunological Tolerance and Absorption Techniques. *J. exp. Med.*, **121**, 439.

GOLD, P. & FREEDMAN, S. O. (1965*b*) Specific Carcinoembryonic Antigens of the Human Digestive System. *J. exp. Med.*, **122**, 467.

HALL, R. R., LAURENCE, D. J. R., DARCY, D., STEVENS, U., JAMES, R., ROBERTS, S. & NEVILLE, A. M. (1972) Carcinoembryonic Antigen in the Urine of Patients with Urothelial Carcinoma. *Br. med. J.*, iii, 609.

HALL, R. R., LAURENCE, D. J. R., NEVILLE, A. M. & WALLACE, D. M. (1973) Carcinoembryonic Antigen and Urothelial Carcinoma. *Br. J. Urol.*, **45**, 88.

LAURENCE, D. J. R., STEVENS, U., BETTELHEIM, R., DARCY, D., LEESE, C., TURBERVILLE, C., ALEXANDER, P., JOHNS, E. W. & NEVILLE, A. M. (1972) The Role of Plasma Carcinoembryonic Antigen in Diagnosis of Gastrointestinal, Mammary and Bronchial Carcinoma. *Br. med. J.*, iii, 605.

LAURENCE, D. J. R. & NEVILLE, A. M. (1972) Foetal Antigens and their Role in the Diagnosis and Clinical Management of Human Neoplasms: A Review. *Br. J. Cancer*, **26**, 335.

NERY, R., BARSOUM, A. L., BULLMAN, H. & NEVILLE, A. M. (1974) Carcinoembryonic Antigen-like Substances of Human Urothelial Carcinomas. *Biochem. J.*, **139**, 431.

NEVILLE, A. M., NERY, R., HALL, R. R., TURBERVILLE, C. & LAURENCE, D. J. R. (1973) Aspects of the Structure and Clinical Role of the Carcinoembryonic Antigen (CEA) and Related Macromolecules with Particular Reference to Urothelial Carcinoma. In Immunology of Malignancy, Eds. M. Moore, N. W. Nisbet and Mary V. Haigh. *Br. J. Cancer*, **28**, Suppl. 1, 198.

SCHOONEES, R., GAMARRA, M. G., MOORE, R. H. & MURPHY, G. P. (1971) The Diagnostic Value of Urinary Cytology in Patients with Bladder Carcinoma. *J. Urol.*, **106**, 693.

THOMPSON, D. M. P., KRUPEY, J., FREEDMAN, S. O. & GOLD, P. (1969) The Radioimmunoassay of Circulating Carcinoembryonic Antigen of the Human Digestive System. *Proc. natn. Acad. Sci. U.S.A.*, **64**, 161.

TURBERVILLE, C., DARCY, D. A., LAURENCE, D. J. R., JOHNS, E. W. & NEVILLE, A. M. (1973) Studies of Carcinoembryonic Antigen (CEA) and a Related Glycoprotein, CCEA-2. Preparation and Chemical Characterization. *Immunochemistry*, **10**, 841.